# Effects of dietary forage to concentrate ratio on nutrient digestibility, ruminal fermentation and rumen bacterial composition in Angus cows

**DOI:** 10.1038/s41598-021-96580-5

**Published:** 2021-08-23

**Authors:** Hao Chen, Chunjie Wang, Simujide Huasai, Aorigele Chen

**Affiliations:** 1 College of Animal Science, Inner Mongolia Agricultural University, Hohhot, 010018 China; 2 College of Veterinary Medicine, Inner Mongolia Agricultural University, Hohhot, 010018 China

**Keywords:** Biochemistry, Developmental biology

## Abstract

This study evaluated effects of dietary forage to concentrate ratio (F:C) on the body weight, digestibility, ruminal fermentation and rumen bacterial composition in Angus cows. Three diets with different F:C (LCD: 65:35, MCD:50:50, and HCD: 35:65) were fed to ninety Angus cows (3.2 ± 0.18 years old, 387.2 ± 22.6 kg). The average daily gain (ADG) and ammonia nitrogen concentration increased (*P* = 0.039 and *P* = 0.026, respectively), whereas the acetate to propionate ratio (*P* = 0.027) and the neutral detergent fiber (NDF) digestibility decreased with increasing concentrate level. The acetate concentration and ruminal pH (*P* = 0.033 and *P* = 0.029, respectively) decreased by feeding HCD diet. Serum amyloid A (SAA), C-reactive protein (CRP), haptoglobin (Hp) and lipopolysaccharide binding protein (LBP) increased under the HCD. The relative abundances of Bacteroidetes, Fibrobacterota, Prevotella and Prevotellaceae UCG-003 decreased, whereas the relative abundances of Ruminococcaceae NK4A214 group, Saccharofermentans and Spirochaetota increased with increasing dietary concentrate level. Our study provides a better understanding of rumen fermentation parameters and microbiota under a wide range of dietary F:C ratios, supporting the potential dietary manipulation of microbes, which could enhance feed digestibility associated with cow rearing.

## Introduction

Nutrition status is one of the main factors influencing the reproductive performance of beef cows^1^, poor nutritional status results in an extended postpartum interval (PPI) and leads to a decreased likelihood of conception^2^. The national research council has concluded that the interval from calving to first estrus and pregnancy rate in healthy beef cows were related to the body condition score at parturition and postnatal nutrition^3,4^.

Houghton et al. found that providing high-energy feedstuffs to cows is necessary to minimize the postpartum interval and optimize conception rates^5^. In practical feedlot industry, feeding high-concentrate diets to ruminants is a common strategy to meet the high energy requirements and improve cost efficiency. A major challenge in the current ruminant feeding systems is how to reconcile feeding of large amounts of cereal grains that support high-production performance with rumen and body health. Compared with low-concentrate diets, high-concentrate diets can be rapidly fermented by ruminal microorganisms to produce short-chain fatty acids (SCFAs), especially propionate and butyrate, which are conducive to enhancing growth performance and improving feed efficiency^6,7^. However, goats fed a diet with 60% concentrate compared to 30% spent less time ruminating during the hours following meal distribution^8^. Moreover, dairy cows fed high-concentrate diets showed lower digestibility of dietary fiber^9^, as was also observed in goats^10^. This result is associated with the accumulation of volatile fatty acids in the rumen, which would reduce buffering capacity and lead to rapid ruminal pH reduction^11^.

Previous studies have shown that prolonged low ruminal pH will lead to increased release of lipopolysaccharides (LPS) in the rumen, which could stimulate the release of proinflammatory cytokines by translocating from the gastrointestinal tract into blood circulation^12,13^. Moreover, high-grain diets also lead to a high incidence of metabolic disorders such as subacute ruminal acidosis (SARA)^14^, laminitis^15^ and fatty liver^16^.

This feeding pattern can also adversely affect both the composition and functionality of rumen microbiota^17^. Ruminants rely on their symbiosis with the microbiota in their rumen, as these microbes allow ruminants to convert nonprotein nitrogen into protein, digest fiber and synthesize vitamins^18,19^. The critical nature of rumen bacterial balance is evident, as influences on rumen health as a consequence of changes in rumen bacterial composition and functionality are involved in the production, health and welfare of ruminants^20,21^. High-grain diets have a potent ability to reshape the rumen bacterial community in terms of altering the microbial composition at the phylum and genus levels, leading to a dysbiotic community and a potential loss of community function^22–24^. Numerous studies have reported that feeding high-grain diets to cattle reduces the richness, evenness, and diversity of the bacteria of the foregut and transforms these microbiotas into a less functional state.

The effects of a high-concentrate diet on rumen fermentation has been widely investigated with respect to the fattening of steers^25,26^, Holstein dairy cows^27^and goats ^28^. However, few comprehensive studies have focused on digestibility, ruminal fermentation and rumen bacterial composition in Angus cows. A better understanding of the ruminal fermentation variation and health status of cows fed diets with different concentrate levels can help to improve their welfare and feed efficiency. Therefore, the objective of this study was to investigate the effects of different forage-to-concentrate ratios on body weight, digestibility, ruminal fermentation and rumen bacterial composition in Angus cows. We hypothesized that the feed digestibility could be improved by increasing dietary concentrate, whereas cows received medium concentrate diet will under a better health status compared with cows receiving high concentrate diet.

## Materials and methods

### Ethics approval and consent to participate

All the animal procedures were carried out according to the protocols approved by the College of Animal Science, Inner Mongolia Agricultural University, China, and we confirm that all methods were carried out in accordance with relevant guidelines and regulations. We confirm that the authors complied with the Animal Research Reporting of In Vivo Experiments (ARRIVE) guidelines^29^.

### Animals, diets and management

Ninety Angus cows (3.2 ± 0.18 years old, 387.2 ± 22.6 kg) with similar initial body weight were assigned to one of three forage to concentrate ratios (F:C): 35:65, 50:50 and 65:35 on a dry matter (DM) basis; the groups were named low concentrate diet (LCD), medium concentrate diet (MCD), and high concentrate diet (HCD), respectively. Corn silage and dry rice straw were used as forage resources. The cows were subjected to a 15-day adaptation period to the experimental diet. Before the adaptation period, cows were fed a basal total mixed ration (TMR) containing 35% concentrate on the farm. In the 15-day adaptation period, cows were then progressively switched from the basal TMR to their experimental TMR by increasing the proportion of the concentrate in the TMR by 5% or 10% every 5 d until the concentrate proportions of the experimental diets were achieved. Cows were individually fed a TMR twice daily at 08:00 and 18:00 h, with free access to diet and water. The ingredients and nutritional composition of the three diets are reported in Table 1.

**Table 1:** Dietary ingredients and chemical composition (g/kg DM).

Item	Group^1^		
	LCD	MCD	HCD
**Ingredient, % of DM**			
Dry rice straw	41.50	26.50	11.50
Corn silage	23.50	23.50	23.50
Corn	18.35	24.87	36.32
Wheat bran	5.33	11.10	10.43
Soybean meal	9.52	12.23	16.45
CaHPO_4_	0.30	0.30	0.30
NaCl	0.50	0.50	0.50
Premix^2^	1.0	1.0	1.0
Total	100	100	100
**Nutrient composition**^**3**^			
OM, % of DM	92.58	92.69	92.47
EE, % of DM	3.35	3.49	3.66
CP, % of DM	12.59	13.61	14.42
ADF, % of DM	25.43	20.70	14.62
NDF, % of DM	48.59	41.56	35.01
NEm,^4^ MJ/kg DM	8.42	9.67	10.91
Ca, % of DM	0.61	0.68	0.63
P, % of DM	0.32	0.35	0.37

^1^LCD, 35:65 concentrate:forage; MCD, 50:50 concentrate:forage; HCD, 65:35 concentrate:forage;

^2^Premix contained (per kg of premix): 420,000 IU of vitamin A, 110,000 IU of vitamin D_3_, 4,200 IU of vitamin E, 2620 mg of Fe, 155 g of Ca, 30 g of P, 820 mg of Cu, 1,800 mg of Mn, 3,500 mg of Zn, 30 mg of I, 35 mg of Se, and 22 mg of Co.

^3^OM, organic matter; EE, ether extract; CP, crude protein; NDF, neutral detergent fiber; ADF, acid detergent fiber; NEm, net energy for maintenance; Ca, calcium; P, phosphorus.

^4^Calculated.

### Apparent total tract nutrient digestibility and feed intake

Following the adaptation period, cows were confined for 60 days. Feed intake data were collected daily. Total mixed ration samples of the 3 experimental diets were collected once per week and stored at − 20 °C to determine nutrient composition. Fecal samples were collected from the rectum from day 55 through day 57 during the trial. For each cow, a 150 g fecal sample was mixed with 20 mL of 10% H_2_SO_4_ and immediately stored at − 20 °C for digestibility determination. The fecal samples were analyzed for dry matter (DM; 934.01), ether extract (EE; 920.39), crude protein (CP; 984.13), ash (942.05), calcium (Ca; 935.13), and total phosphorus (TP; 946.06) according to AOAC methods^30^. The NDF and ADF were analyzed following the methods^31^, NDF was determined with heat-stable amylase and sodium sulphite addition and expressed inclusive of residual ash, and ADF was determined and expressed inclusive of residual ash. Acid-insoluble ash was assayed using the method described by Van Keulen and Young ^32^. The equation used for digestibility calculation was: ND (%) = 100 − (A/B × C/D) × 100, where ND = nutrient digestibility, A = AIA content in feed, B = AIA content in feces, C = nutrient content in feces, and D = nutrient content in feed.

### Blood sampling and analysis

Six cows are randomly selected from each group, and blood samples are collected from these animals each time. Blood samples (15 mL) were collected by jugular venipuncture into heparinized tubes before the morning feed allowance on days 20, 40 and 60. They were immediately centrifuged at 3,000 × *g* for 15 min at 4 °C to harvest plasma, which was stored at − 20 °C for further analysis. The concentrations of plasma interleukin-1β (IL-1β), interleukin-6 (IL-6), interleukin-8 (IL-8) and tumor necrosis factor-α (TNF-α), lipopolysaccharide binding protein (LBP), haptoglobin (Hp), serum amyloid A (SAA) and C-reactive protein (CRP) were determined by bovine ELISA kits (Beijing Sino-uk Institute of Biological Technology) according to the manufacturer's instructions. The LPS concentrations in the rumen fluid and plasma were determined using an ELISA kit purchased from Shanghai Sinobest Biotechnology Co., Ltd. (Shanghai, China) according to the manufacturer’s instructions.

### Ruminal sampling and analysis

Rumen fluid samples were collected from the 18 cows (consistent with blood sample collection). Ruminal fluid samples were collected 3 h after the morning feeding on day 60, as described methods, ruminal fluid samples were collected by an esophageal tube equipped with a strainer and a syringe, to avoid contamination by saliva, the initial rumen fluid collected was discarded, approximately 200 mL of rumen fluid was collected of each cow^34^. Rumen fluid samples were detemined immediately for the pH value. Then, the filtrate was centrifuged at 10,000 × *g* for 15 min. In addition, approximately 10 mL of rumen fluid samples were acidified with 2.5 mL of 25% HPO_3_ for the quantification of NH_3_-N^35^. The quantitation of VFAs was performed as described in a previous study. The quantitation of VFAs was performed according to Tian et al^33^^.^ The remaining ruminal fluid samples were transferred into sterile and pyrogen-free centrifuge tubes, and was immediately frozen in liquid nitrogen and stored at − 80 °C for further genomic DNA extraction.

### DNA Extraction, 16S rRNA Gene Amplicon and Sequencing

After bead-beating and incubating at 90 °C for 10 min, total ruminal fluid DNA was extracted from the ruminal fluid samples using the E.Z.N.A.® soil DNA Kit (Omega Bio-tek, Norcross, GA, U.S.) according to manufacturer’s instructions. The DNA extract was checked on 1% agarose gel, and DNA concentration and purity were determined with NanoDrop 2000 UV–vis spectrophotometer (Thermo Scientific, Wilmington, USA), and the protocol has been previously described in detail^36^. The hypervariable region V3-V4 of the bacterial 16S rRNA gene were amplified with primer pairs 338F (5'-ACTCCTACGGGAGGCAGCAG-3') and 806R(5'-GGACTACHVGGGTWTCTAAT-3')^34^. The PCR amplification of 16S rRNA gene was performed as follows: initial denaturation at 95 °C for 3 min, followed by 27 cycles of denaturing at 95 °C for 30 s, annealing at 55 °C for 30 s and extension at 72 °C for 45 s, and single extension at 72 °C for 10 min, and end at 4 °C^33^. The PCR mixtures contain 5 × TransStart FastPfu buffer 4 μL, 2.5 mM dNTPs 2 μL , forward primer (5 μM) 0.8 μL, reverse primer (5 μM) 0.8 μL, TransStart FastPfu DNA Polymerase 0.4 μL, template DNA 10 ng, and finally ddH2O up to 20 μL. PCR reactions were performed in triplicate^33^. The PCR product was extracted from 2% agarose gel and purified using the AxyPrep DNA Gel Extraction Kit (Axygen Biosciences, Union City, CA, USA) according to manufacturer’s instructions and quantified using Quantus™ Fluorometer (Promega, USA). Purified amplicons were pooled in equimolar and paired-end sequenced on an Illumina MiSeq PE300 platform/NovaSeq PE250 platform (Illumina, San Diego,USA) according to the standard protocols^37^.

The raw 16S rRNA gene sequencing reads were demultiplexed, quality-filtered by fastp version 0.20.0 and merged by FLASH version 1.2.7 with the following criteria: (i) the 300 bp reads were truncated at any site receiving an average quality score of < 20 over a 50 bp sliding window, and the truncated reads shorter than 50 bp were discarded, reads containing ambiguous characters were also discarded^38^; (ii) only overlapping sequences longer than 10 bp were assembled according to their overlapped sequence. The maximum mismatch ratio of overlap region is 0.2. Reads that could not be assembled were discarded^34^; (iii) Samples were distinguished according to the barcode and primers, and the sequence direction was adjusted, exact barcode matching, 2 nucleotide mismatch in primer matching^38^.

Operational taxonomic units (OTUs) with 97% similarity cutoff were clustered using UPARSE version 7.1^39^, and chimeric sequences were identified and removed^40^. The taxonomy of each OTU representative sequence was analyzed by RDP Classifier version 2.2 against the 16S rRNA database (Silva v138) using confidence threshold of 0.741.

### Statistical analysis

Significant analyses was performed by using SPSS software (SPSS v. 21, SPSS Inc.; Chicago, IL, USA). Significance analyses of the ADG, DMI, plasma concentration, pH and rumen fermentation parameters in diets with different dietary F:C ratios were conducted by using one-way ANOVA in SPSS. Significant differences are presented at the level of *P* < 0.05.

## Results

### Dry matter intake, average daily gain and apparent nutrient digestibility

DMI, ADG and apparent nutrient digestibility data are shown in Table 2. DMI and DM digestibility increased with increasing concentrate proportion in the diet (*P* = 0.039 and *P* = 0.015, respectively). The ADG in the LCD group was the lowest among the experimental diets (*P* = 0.018). Compared with the LCD group, the HCD group had increased digestibility of OM and CP (*P* < 0.001) . Moreover, the HCD group had decreased (*P* = 0.036) the digestibility of NDF relative to the other groups.

**Table 2:** Effect of different dietary levels of concentrate on intake, average daily gain and digestibility in Angus cows.

Items	Diets^1^			
	LCD	MCD	HCD	*P*-value
Dry matter intake (DMI), kg/d	8.36 ± 0.26^c^	9.51 ± 0.22^b^	10.59 ± 0.31^a^	0.039
Average daily gain, kg/d	0.74 ± 0.092^b^	1.03 ± 0.017^a^	1.12 ± 0.075^a^	0.018
Nutrient digestibility, %				
Dry matter	62.5 ± 2.86^c^	68.3 ± 1.61^b^	73.0 ± 3.74^a^	0.015
Organic matter	59.1 ± 3.81^b^	63.4 ± 2.93^ab^	67.3 ± 4.76^a^	< 0.001
Crude protein	53.5 ± 1.09^c^	57.3 ± 0.86^ac^	62.7 ± 1.17^a^	< 0.001
Ether extract	59.0 ± 3.72	57.2 ± 2.33	61.0 ± 3.82	0.426
Acid detergent fiber	64.6 ± 5.78	64.2 ± 2.69	62.8 ± 2.01	0.581
Neutral detergent fiber	66.9 ± 2.07^a^	63.1 ± 3.53^a^	58.5 ± 1.66^b^	0.036

^a,b,c^Means bearing different superscripts in the same row differ significantly (*P* < 0.05).

^1^LCD, 35:65 concentrate:forage; MCD, 50:50 concentrate:forage; HCD, 65:35 concentrate:forage;

### Ruminal fermentation

As shown in Table 3, compared with the LCD group, the HCD and MCD groups had increased ammonia nitrogen (*P* = 0.026), butyrate (*P* < 0.001) and isovalerate (*P* = 0.016) concentrations in ruminal fluid. Moreover, the HCD group had increased (*P* = 0.021) propionate concentrations and decreased acetate (*P* = 0.033) concentrations in ruminal fluid relative to those of the LCD group. The acetate to propionate ratio decreased (*P* = 0.027) with the increasing concentrate proportions in the diet. The HCD group had decreased (*P* = 0.029) ruminal pH relative to the other groups.

**Table 3:** Effect of different dietary levels of concentrate on rumen fermentation in Angus cows.

Items	Diets^1^			
	LCD	MCD	HCD	P
Ammonia nitrogen (mg/dL)	8.39 ± 0.86^b^	10.67 ± 1.15^a^	11.41 ± 0.67^a^	0.026
Acetate	62.69 ± 5.82^a^	57.27 ± 9.13^ab^	54.11 ± 4.21^b^	0.033
Propionate	12.87 ± 1.26^b^	15.36 ± 1.75^ab^	18.15 ± 2.81^a^	0.021
Butyrate	7.56 ± 0.50^b^	12.15 ± 0.69^a^	13.68 ± 1.33^a^	< 0.001
isobutyric acid	0.55 ± 0.09	0.59 ± 0.06	0.62 ± 0.12	0.819
Valerate	1.72 ± 0.13^b^	1.96 ± 0.08^b^	2.38 ± 0.17^a^	0.375
Isovalerate	1.76 ± 0.03^b^	2.14 ± 0.09^a^	2.27 ± 0.05^a^	0.016
Total volatile fatty acids (mmol/L)	87.25 ± 8.57	89.47 ± 10.22	91.21 ± 4.35	0.623
Acetate to propionate ratio (A:P)	4.87 ± 0.03^a^	3.73 ± 0.02^b^	2.98 ± 0.04^c^	0.027
pH	6.62 ± 0.21^a^	6.57 ± 0.19^a^	6.08 ± 0.32^b^	0.029

^a,b,c^Means bearing different superscripts in the same row differ significantly (*P* < 0.05).

^1^ LCD, 35:65 concentrate:forage; MCD, 50:50 concentrate:forage; HCD, 65:35 concentrate:forage;

### Inflammatory response

As shown in Table 4, plasma acute phase proteins (APPs) were increased by high dietary concentrate level. Compared with the LCD and MCD groups, the HCD group had increased CRP (*P* = 0.036), SAA (*P* < 0.001), Hp (*P* < 0.001) and LPS (*P* < 0.001) concentrations in plasma. Moreover, the HCD group had increased LBP (*P* = 0.011) concentrations in plasma relative to the LCD group.

**Table 4:** Effect of different dietary levels of concentrate on blood parameters in Angus cows.

Items	Diets^1^			
	LCD	MCD	HCD	*P*-value
CRP mg/L	8.18 ± 0.21^b^	8.43 ± 0.19^b^	11.05 ± 0.57^a^	0.014
Hp ng/mL	179.6 ± 12.60^b^	194.09 ± 17.82^b^	271.67 ± 32.19^a^	< 0.001
SAA ug/mL	5.53 ± 0.25^b^	6.09 ± 0.22^b^	7.28 ± 0.38^a^	< 0.001
LBP μmol/L	143.24 ± 19.67^b^	151.52 ± 18.53^ab^	195.65 ± 27.29^a^	0.011
Ruminal LPS EU/mL	19,824.67 ± 1529.63^c^	23,537.32 ± 1670.11^b^	36,183.28 ± 2432.28^a^	< 0.001
Plasma LPS EU/mL	0.23 ± 0.02^b^	0.28 ± 0.03^b^	0.49 ± 0.06^a^	< 0.001

CRP = C-reactive protein; Hp = haptoglobin; SAA = serum amyloid A; LBP = LPS-binding protein; ruminal LPS = lipopolysaccharide in ruminal fluid; plasma LPS = plasma lipopolysaccharide;

^a,b,c^Means bearing different superscripts in the same row differ significantly (*P* < 0.05).

^1^LCD, 35:65 concentrate:forage; MCD, 50:50 concentrate:forage; HCD, 65:35 concentrate:forage.

### Bacterial abundance and diversity analysis

**Figure 1:**
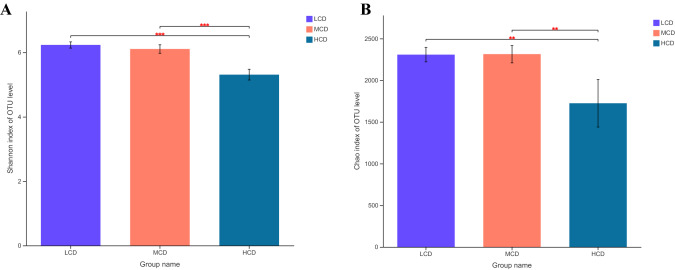
Differences in ruminal bacterial diversity and richness between cows fed diets with different forage to concentrate ratios. Bacterial diversity was estimated by Shannon index (**A**). Bacterial richness estimated by the Chao1 value (**B**). LCD, 35:65 concentrate:forage; MCD, 50:50 concentrate:forage; HCD, 65:35 concentrate:forage; Asterisks indicate significant difference between the groups (P < 0.01).

A total of 948,462 quality-checked sequences were obtained from all 18 samples, and 48,331—63,456 sequences were returned for each sample. After OTU picking and chimera checking, a total of 2,959 OTUs were calculated for all the samples at 3% dissimilarity. Good’s coverages for all samples exceeded 99%, which indicated the accuracy and reproducibility of the sequencing. According to the Chao1 value and Shannon index, the HCD group supported significantly less diversity (*P* < 0.01) than the LCD group based on the Shannon index (Fig. 1A). Chao1 value analysis indicated a similar tendency of richness (Fig. 1B).

**Figure 2:**
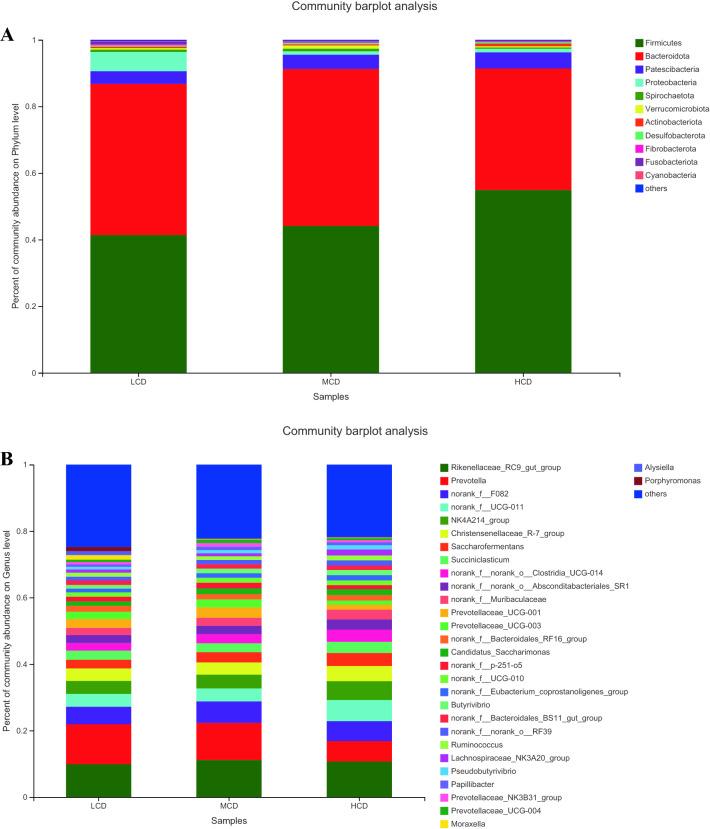
Phylum-level (**A**) and genera-level (**B**) composition of the rumen microbiome. Color-coded bar plot showing the average bacterial phylum or genera distribution across the different groups that were sampled. LCD, 35:65 concentrate:forage; MCD, 50:50 concentrate:forage; HCD, 65:35 concentrate:forage;

Taxonomic analysis of the reads revealed the presence of 22 bacterial phyla. Among these phyla, Bacteroidetes and Firmicutes had relatively high abundances, with mean abundance levels of 47.2 ± 3.1% (mean ± standard error of the mean) and 42.3 ± 2.7%, respectively (Fig. 2A). At the genus level, 216 genera were identified in the cow rumen samples. The predominant genera were the Rikenellaceae RC9 gut group (10.52 ± 1.2%), Prevotella (9.78 ± 1.6%), the Christensenellaceae R-7 group (2.43 ± 0.6%), unclassified Ruminococcaceae (4.67 ± 0.8%), Prevotellaceae UCG-003 (3.16 ± 0.3%) and the Ruminococcaceae NK4A214 group (4.52 ± 0.6%) (Fig. 2B).

**Figure 3:**
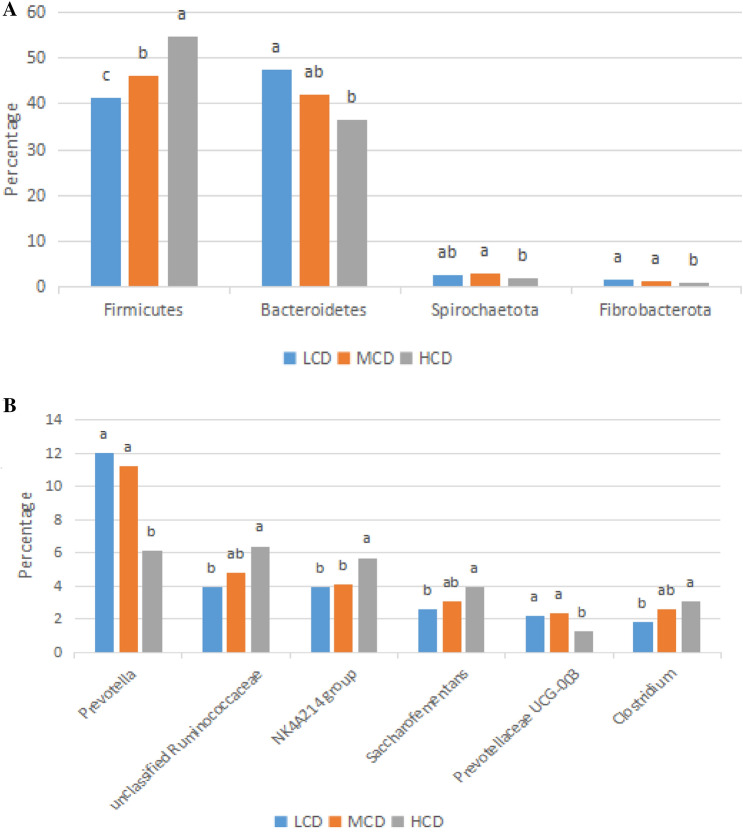
Changes in the relative abundance of (**A**) the most dominant phylum and (**B**) the most dominant genera in the rumen microbial community of cows with different F:C ratios diets. Boxes with a different letter are significantly different at P < 0.05 or P < 0.1 (Firmicutes) by t-test analyses. LCD, 35:65 concentrate:forage; MCD, 50:50 concentrate:forage; HCD, 65:35 concentrate:forage.

With increasing dietary concentrate levels, we observed a profound change in rumen microbial composition at the phylum and genus levels (Fig. 3A). The dietary concentrate level showed a statistically significant effect on the relative abundance of Firmicutes (*P* = 0.003), which increased with dietary concentrate level and became the most abundant phylum in samples from the HCD group. By contrast, the relative abundances of Bacteroidetes and Fibrobacterota decreased with dietary concentrate level and were less abundant (*P* = 0.028 and *P* = 0.012, respectively) in samples from the HCD group compared with the LCD group. Spirochaetota was decreased (*P* = 0.035) in the HCD group. In this study, we found that the relative abundances of genera were also affected by dietary concentrate levels (Fig. 3B). With increasing dietary concentrate levels, the relative abundances of unclassified Ruminococcaceae, Ruminococcaceae NK4A214 group, Saccharofementans, and Clostridium significantly increased; however, the relative abundance of Prevotella and Prevotellaceae UCG-003 declined (*P* = 0.029 and *P* = 0.007, respectively).

## Discussion

It is generally accepted that changing the level of concentrate in ruminant diets can significantly affect the DMI and nutrient digestibility^42^. In the present study, the DMI increased significantly with increasing concentrate levels. This may be due to the high content of structural carbohydrates such as cellulose and hemicellulose in roughage, which reduces the flow rate of chyme in the digestive tract of beef cattle and inhibits feed intake^43^. Increasing the proportion of concentrate can effectively improve the palatability of the diet and accelerate the flow rate of chyme, which effectively increases the feed intake of beef cattle^44^. However, a high-concentrate diet reduced the DMI of fattening beef cattle^45^. This may have been due to the high body fat content of fattening beef cattle; scholars have observed a close negative correlation between body fat content and DMI in beef cattle^46^, and the research objects in this experiment were lean cows with low body fat. The difference in body fat of beef cattle between the two experiments may explain the difference in the effect of the high-concentrate feed on the DMI.

In line with the increase in DMI, the intake of digestible energy, organic matter and crude protein increased with increasing dietary concentrate levels, while the intake of NDF and ADF decreased. This is due to the increase in DMI, and the intake of nutrients is determined by the DMI. The contents of digestible energy, OM and CP in the concentrate were higher than those in the roughage, while the contents of NDF and ADF were lower. Therefore, with the increase in dietary concentrate level, the nutrient intake of experimental cows in this study showed the above changes. Concentrates can provide more nutrients for the growth of rumen microorganisms and promote rumen fermentation. Therefore, increasing concentrate levels in the diet usually has a positive effect on nutrient digestibility. Consistent with the previous results^47^, the significantly increased digestibility of DM, OM and CP was present in cows fed high-concentrate diet. In recent years, similar results from goats and buffalos have been reported^48,49^. The reason for improving the nutrient digestibility of beef cattle by increasing the concentrate level may be that the concentrate contains a large amount of nonfibrous carbohydrates, which can be rapidly fermented by rumen microorganisms, thus improving nutrient digestibility. According to the current study, digestibility of NDF in the HCD group was significantly lower than that in the LCD and MCD groups. As previously observed, the decrease in rumen pH caused by a high grain diet could reduce the digestibility of NDF^50^. The current study indicates that the rumen pH of the HCD group was significantly lower than that of the other two groups. In this study, the ADG of cows increased significantly with the increase of dietary concentrate level, and the increase of DMI partly explained the reason for the increase of daily gain. In addition, a large number of nonfibrous carbohydrates in the concentrate could be rapidly fermented by rumen microorganisms to produce a large number of SCFAs and improve the nutrient digestibility, which had a positive effect on the improvement of growth performance of beef cattle.

pH value, NH_3_-N levels and VFA concentration are the main internal environmental indicators of rumen fermentation^51^. In this study, increases in the dietary concentrate level significantly decreased the ruminal pH, which might be due to greater starch intake. The pH for cows fed the low F:C diet (pH = 6.08) in the present study compared with cows fed barley-based diets (pH < 5.8) also confirms the lower risk of subacute ruminal acidosis for dairy cows fed corn- versus barley-based diets because of the lower digestion rate of corn starch^52^. In our study, increases in the dietary concentrate level did not influence the total volatile fatty acid concentration, which could be due to the ability of the rumen system to adapt to changing dietary concentrate level through the self-adjustment of rumen microorganisms. Furthermore, HCD diets reduced acetate concentrations but significantly increased the propionate proportions, thereby resulting in a significant reduction in the A:P ratio. Consistent with many previous studies^14,48,53^, our study confirmed that feeding a HCD diet shifted the fermentation pattern from acetate to propionate. A decrease in the A:P ratio reflects an improvement in the feed energy utilization efficiency^54^. This could explain the higher growth performance observed in the current experiment, since propionate and butyrate are conducive to enhancing growth performance. The ruminal NH_3_-N concentration is mainly determined by the rate of nitrogen decomposition and ammonia absorption and utilization by rumen microorganisms^55^. In the current study, HCD diets elevated NH_3_-N concentrations, which is consistent with the former study^26^. The higher ruminal NH_3_-N concentration might be explained by the intake of nitrogen being greater with low versus high F:C diets.

Lipopolysaccharides are the degradation products of rumen bacteria, as many microbial species are not able to survive at low pH and undergo widespread lysis^56^. Yang et al. indicated that the ruminal LPS concentrations of beef steers increased with an increase in concentrate in the diet^57^. In the current study, markedly greater concentrations of rumen LPS were observed with increasing dietary grain levels, which is consistent with the previous research^58^. A lower ruminal pH can result in greater death of gram-negative bacteria and the excessive release of LPS. Previous research showed that LPS could be translocated from the rumen epithelium into the blood and ultimately induce inflammatory responses in the body^15^, such as increased proinflammatory cytokine release and APP production^16,32^. Moreover, Gozho et al.^59^ reported that a high-grain diet led to a significant increase in rumen fluid LPS concentration as well as plasma concentrations of SAA and Hp, which was observed in the HCD groups in the current study. Hepatic and Kupffer cells could breakdown LPS and subsequently be excreted^60^. In this study, we did not detect an increasing LPS concentration in cows from MCD compared with LCD, which may be due to the clearing capacity of the liver cells, and the translocated LPS was degraded in the liver^18^.

With increasing dietary concentrate levels, the richness and diversity of the ruminal bacteria deeply decreased, as recorded by other authors where high-grain diets were fed to cattle^55,61^. This finding might be due to the higher level fermentable substrate present in the MCD and HCD diets. Firmicutes and Bacteroidetes were the most abundant phyla in digesta of the ruminal fluid of cattle; together, they represented between 76.0 and 96.1% of bacteria in these digesta^12,54^. In the current study, Firmicutes and Bacteroidetes were the dominant phyla, accounting for approximately 90% of the total bacterial population, and were affected by dietary treatment. Previous studies observed a decrease in Bacteroidetes and an increase in Firmicutes in the rumen after excessive grain feeding^62^. In contrast, Hua et al.^63^ found that these two phyla were not affected by dietary treatment, which may be due to the susceptibility of ruminants and the resilience of the bacteria to dietary changes. Compared with the members of the phylum Firmicutes, the members of phylum Bacteroidetes had higher mean glycoside hydrolase (GH) and polysaccharide lyase (PL) genes per genome as well as a greater range of GHs and PLs, so the phylum Bacteroidetes is the primary degrader of complex polysaccharides in the plant cell wall^64^. As ruminants rely on GHs and PLs for efficient degradation and digestion of fiber, a high-concentrate diet leads to an undesirable increase in the Firmicutes to Bacteroidetes ratio in the digesta^55^.

In grain-fed cattle, the relative abundances of the phylum Fibrobacter were decreased in the microbial communities in the rumen^20^. In this study, the relative abundance of Fibrobacter decreased in the ruminal fluid of the HCD group compared with the other groups and resulting in an increase in the proportion of Firmicutes. Furthermore, the phylum Fibrobacteres is one of the major contributors to the free LPS pool in the rumen digesta, which may explain the higher LPS content in HCD group. High-grain feeding can also alter populations of pathogenic bacteria. Clostridium is a ubiquitous genus in the gastrointestinal tract, and some members of Clostridium sensu stricto are generally perceived as pathogenic^65,66^. In previous studies, the populations of pathogenic Escherichia coli and Clostridium perfringens in the rumen were commonly found to be associated with high-grain feeding^7,21^. In accordance with other studies, the abundance of Clostridium sensu stricto was increasing in the HCD cows, which might adversely affect ruminal health, although further investigation is needed. It is worth attention that in addition to these changes in classified bacteria, unclassified Ruminococcus was also significantly affected by the dietary treatments. The precise role of the unclassified bacteria is not clear, but they might have vital roles in nonstructural carbohydrate digestion.

It is acknowledged that Prevotella is the most abundant genus of Bacteroidetes, and can use a variety of substrates in the rumen^7^. In the present study, the abundance of the genus Prevotella were negatively correlated with the dietary concentrate level, which is in accordance with previous studies demonstrating that a high-grain diet lowers the relative abundance of Prevotella^6,62^. Although the relationship between the substrate and growth of Prevotella is not clear, a decrease in pH may affect the relative abundance of Prevotella. Previous studies have proved that Prevotellaceae UCG-003 was involved in glucose metabolism, producing acetic acids as the major fermentation end-products^67,68^. Thus, it makes sense that the genus Prevotellaceae UCG-003 presented at a higher level in the LCD group. A recent study showed that Ruminococcaceae NK4A214 was positively correlated with isobutyrate and isovalerate concentrationsyy^51^. In the present study, with the higher proportion of the genus Ruminococcaceae NK4A214 in the HCD group, which could explain the lower isovalerate concentration in the LCD group.

## Conclusions

In summary, our results clearly show that the different dietary F:C ratios affected the growth performance, rumen fermentation and blood parameters of Angus cows. Increasing the dietary concentrate feed level from 35 to 65% exerted a positive effect on the DMI, BW gain, and gain rate of Angus cows. Moreover, low F:C ratios significantly increased ammonium nitrogen, reduced the A:P molar ratio and changed rumen fermentation from acetate fermentation to propionate fermentation. Angus cows fed 65% concentrate for barn feeding can result in higher LPS concentrations in ruminal fluid and plasma, which may adversely affect health status due to an increased risk of subacute ruminal acidosis. These findings support the potential for microbial manipulation by diet, which could enhance feed digestibility and health status associated with cow rearing.

## Data Availability

The data sets used and/or analyzed during the current study are available from the corresponding author on reasonable request.
